# Targeted regulation of FoxO1 in chondrocytes prevents age‐related osteoarthritis via autophagy mechanism

**DOI:** 10.1111/jcmm.17319

**Published:** 2022-05-13

**Authors:** Jiaji Yue, Aikebaier Aobulikasimu, Weichao Sun, Shuyu Liu, Wei Xie, Wei Sun

**Affiliations:** ^1^ Department of Bone and Joint Surgery Shenzhen Second People's Hospital The First Affiliated Hospital of Shenzhen University Shenzhen China

**Keywords:** autophagy, chondrocytes, FoxO1, osteoarthritis

## Abstract

Autophagy is designated as a biological recycling process to maintain cellular homeostasis by the sequestration of damaged proteins and organelles in plasma and cargo delivery to lysosomes for degradation and reclamation. This organelle recycling process promotes chondrocyte homeostasis and has been previously implicated in osteoarthritis (OA). Autophagy is widely involved in regulating chondrocyte degeneration markers such as MMPs, ADAMSTs and Col10 in chondrocytes. The critical autophagy‐related (ATG) proteins have now been considered the protective factor against late‐onset OA. The current research field proposes that the autophagic pathway is closely related to chondrocyte activity. However, the mechanism is complex yet needs precise elaboration. This review concluded that FoxO1, a forkhead O family protein, which is a decisive mediator of autophagy, facilitates the pathological process of osteoarthritis. Diverse mechanisms regulate the activity of FoxO1 and promote the initiation of autophagy, including the prominent AMPK and Sirt‐2 cellular pathways. FoxO1 transactive is regulated by phosphorylation and acetylation processes, which modulates the downstream ATGs expression. Furthermore, FoxO1 induces autophagy by directly interacting with ATGs proteins, which control the formation of autophagosomes and lysosomes fusion. This review will discuss cutting‐edge evidence that the FoxO–autophagy pathway plays an essential regulator in the pathogenesis of osteoarthritis.

Osteoarthritis is a widespread and incapacitating disease, with 250 million people are currently estimated affected.^1^ With the striking impacts of ageing and the rapidly expanding population in obesity worldwide, osteoarthritis exerts a substantial medical burden for the patients and healthcare systems.^2^, ^3^ Osteoarthritis (OA) has gradually become one of the primary diseases that affect patients’ quality of life due to its high incidence and disability rate. Along with increasing quantities in sports injuries, the syndromic condition is becoming more socioeconomic burdensome.^4^

The data reported by the Lancet^4^ show that the incidence of knee OA in people over 50 years old is about 14%–38% in Asia, 23% in Europe and about 19% in North America. Meanwhile, the consumption of medical resources accounts for 1%–2.5% of GDP in developed countries. At the same time, the indirect losses, including the decline in quality of life, the economic burden in medical rehabilitation and unemployment, are even more magnificent.^2^ The WHO data reveal that, by 2015, the years lived with disability (YLD) caused by OA accounted for approximately 3.9% of all diseases globally. By 2020, OA will become the fourth‐largest cause of YLD loss.^5^ Therefore, research in the pathogenesis and progression mechanism of OA disease and striving to explore feasible solutions and targets for treating or delaying the progression of the disease has significant clinical value and social significance.

## 1 AGEING AND SENESCENCE‐RELATED CHONDROCYTE APOPTOSIS IS THE CRITICAL DETERMINANT IN THE PATHOGENESIS OF OSTEOARTHRITIS

The aetiology of OA is complex and needs elaboration. Evidence shows a significant correlation between increased susceptibility to OA and ageing.^4^ In the early stage of OA progression, ageing promotes articular cartilage degeneration, elastic cartilage gradually degrades into fibrocartilage, and its cushioning and stabilizing effect on joints gradually declines. Cartilage fibrosis increases the compactness of collagen and impairs the microenvironment of chondrocytes. The hypertrophic pathogenesis changes such as morphology alteration and osteogenic differentiation are induced. Chronic injury‐induced bleeding can cause aseptic inflammation of the synovium, chondrocytes and subchondral bone tissue in the articular cavity, thereby accelerating the destruction of the microenvironment and the degeneration of the extracellular cartilage matrix and ultimately leading to the initiation of chondrocytes apoptosis. Oxidative stress damage caused by ageing accumulates in chondrocytes.^5^ By interfering with cellular autophagy, the progress of apoptosis is stimulated, and ultimately causing chondrocytes and cartilage matrix to be replaced by osteoblasts and bone tissue. The pathogenesis alteration eventually induces membrane hyperplasia; inflammatory factors thrive in synovial fluid, cartilage calcification, subchondral bone sclerosis and various OA‐specific pathological manifestations.

**FIGURE 1:**
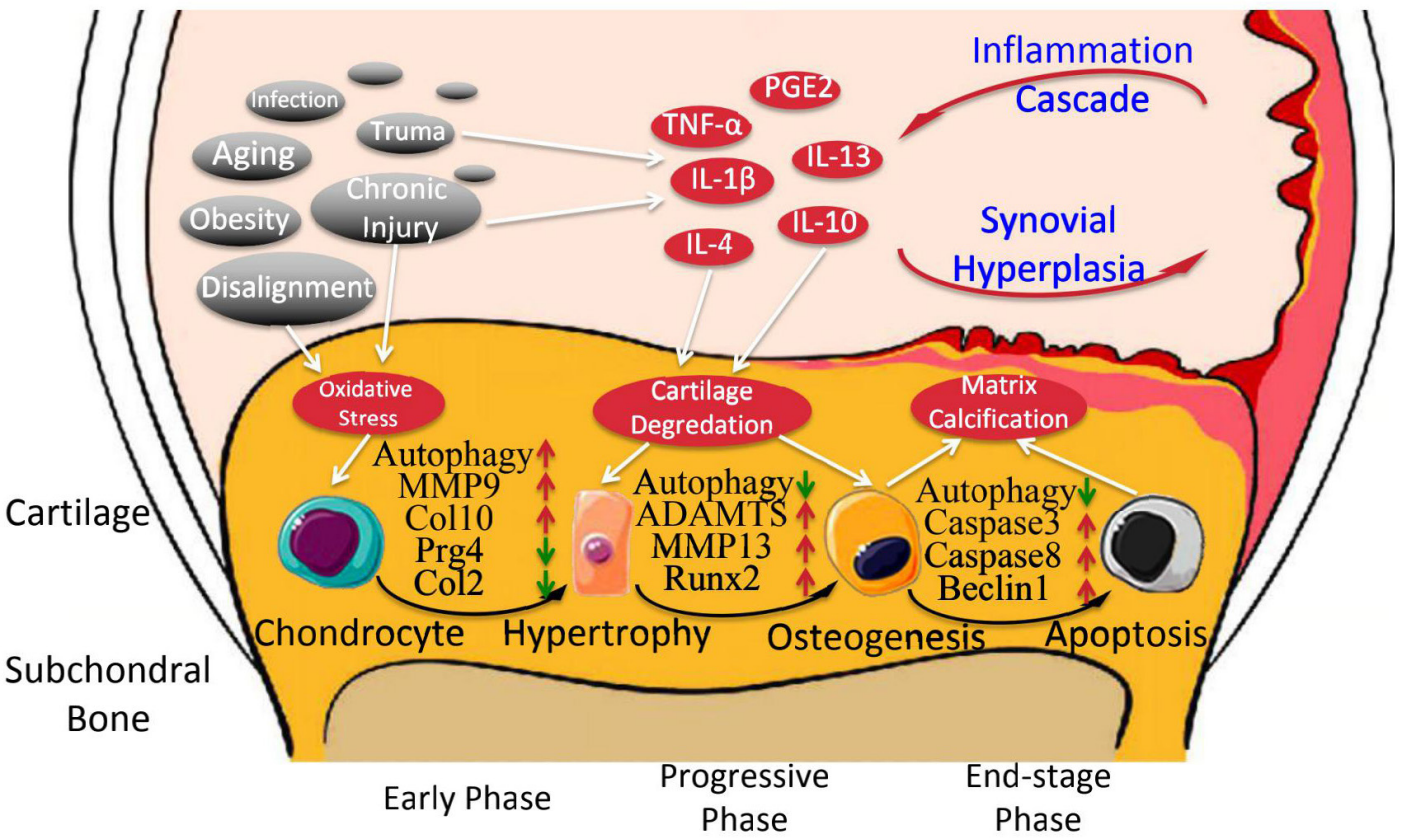
In the progression of OA, chondrocytes undergo a series of pathological alterations of homeostasis impairment‐hypertrophy‐osteogenic differentiation‐apoptosis and finally trigger the OA phenomenon. In the early stage of OA, autophagy activation can effectively prevent chondrocyte hypertrophy (MMP9, Col10 increase) and chondrocytes marker (Prg4, Col2α1) decrease. With the deterioration of OA, the level of autophagy continues to decrease, leading to the osteogenesis markers (ADAMTS, MMP13 and Runx2) and apoptosis markers (Caspase3, Caspase8 and Beclin1) increase and eventually resulting in the cartilage matrix calcification. Ageing and chronic damage are the main initiating factors of OA and directly or indirectly activate inflammatory factors inducing stress damage to chondrocytes. Inflammatory factors also aggravate the joints by synovial hyperplasia and inflammation intensification

As an essential factor in stabilizing articular cartilage homeostasis, chondrocytes undergo a series of alterations in the OA progression. Disequilibrate of chondrocyte homeostasis by impairment causes the chondrocytes hypertrophy and further promotes osteogenic differentiation, eventually inducing cartilage degradation and apoptosis of chondrocytes.^6^ In the pathogenesis of degeneration, the expression of matrix metalloproteinases (MMP9 and MMP13) and various cartilage matrix proteases, such as ADAMTS5 and ADAMTS7, can enzymolysis the type II collagen (Col2α1) and proteoglycan (aggrecan, Acan) in the cartilage matrix. It produces decomposition, which further triggers the degeneration of the cartilage matrix and the transformation into a bone matrix.^7^ In the early stage of OA, autophagy‐mediator LC3‐II, beclin‐1 and ATG5 in articular chondrocytes increased as the feedback reaction to homeostasis impairment. The expression of matrix proteases, such as MMP9, MMP13 and ADAMTS5, ADAMTS7 is inhibited, the metabolic decomposition of Acan, Col2α1, and other multiple helix collagen protects cartilage is prevented. However, with the progress of OA disease, oxidative stress damage continues to accumulate, and the level of autophagy‐mediated by LC3‐II, beclin‐1 and ATG5 in chondrocytes gradually decreases, and it promotes the hypertrophy marker of chondrocytes such as Runx2, Col10α1 and MMP9. The increase in osteogenic differentiation markers leads to hypertrophy of chondrocytes and further differentiation into osteoblasts, which eventually triggers the activation of caspase3 and ccaspase9 and initiates the apoptosis program^8^ (Figure 1). In addition, as the content of elastic fibres in cartilage decreases, the increase in mechanical stress will also cause the reduction in the expression of ULK1, LC3‐II and beclin‐1 in chondrocytes, further accelerating the apoptosis of chondrocytes.^9^

## 2 AUTOPHAGY IS AN ESSENTIAL REGULATOR FOR CHONDROCYTE APOPTOSIS AND THE PATHOGENESIS OF OA

Studies have reported that autophagy plays a significant role in the biological development of bone and cartilage and maintaining physiological homeostasis. On the contrary, ageing exerts an imbalance in autophagy and causes deterioration in chondrocytes apopotosis.^10^ Developmental biology studies in mice have revealed that fibroblast growth factor 18 (FGF‐18) activates JNK1 through the fibroblast growth factor receptor 4 (FGFR‐4) signalling pathway, which activates the autophagy‐related protein 7 (ATG 7) and the downstream autophagy‐mediated type II collagen secretion in chondrocytes.^11^ In the ageing mice model, chondrocytes showed a significant decrease in the number of autophagosomes before the pathological alteration of OA. Meanwhile, a reduction in the ATG5 and LC3 and an increase in the apoptosis marker poly ADP‐ribose polymerase (PARP) reveal the impairment of chondrocyte homeostasis. With the gradual aggravation of ageing, the level of LC3‐II in chondrocytes decreases progressively, and the expression of MMP13 increases progressively, all of which continue to indicate a correlation of autophagy inhibition and OA progression.^12^

The ATG5 knockout mouse model indicated that the gene and protein levels of Col2α1 and Acan decreased significantly, while the expression of matrix protease MMP13 and ADAMTS5 increased.^13^ This study also reflects that autophagy in chondrocytes functions as the protector of cartilage degradation.^13^ The sirolimus or rapamycin inhibits the mammalian target of rapamycin (mTOR) signalling pathway and significantly increases the expression of Col2α1, Acan and Sox9 in ATDC5 chondrocytes. The catabolism markers Col10α1, MMP9, MMP13 and ADAMTS5 and the apoptosis markers caspase3, caspase9, beclin‐1 and beclin‐2 significantly reduced.^11^ The above‐mentioned evidence further confirms that autophagy is important in maintaining chondrocytes and cartilage matrix homeostasis and inhibiting chondrocyte apoptosis. The histological study of clinical cases also found similar results, and mTOR expression increased in joint samples of patients with OA. The specific ligand rapamycin can bind to the intracellular receptor FKBP‐12 to form a complex and act directly on mTOR. The FRB domain (FKBP‐12‐rapamycin binding site) can effectively inhibit its protein activity and increase the level of autophagy. Therefore, regulating the mTOR signalling pathway of autophagy upstream can effectively reduce the destruction of the cartilage matrix.^14^, ^15^ Similarly, the mTOR knockout mouse model reveals that autophagy was significantly activated in chondrocytes, effectively reducing chondrocyte apoptosis and maintaining the homeostasis of the articular cartilage microenvironment. Articular cartilage degeneration decelerated in the surgical‐induced OA mouse model.^16^ On the contrary, chondrocyte‐targeted ATG5 and ATG7 knocked out mouse model reveals the accumulation in caspase‐mediated apoptosis and acceleration of OA disease progression.^17^

## 3 FoxO1 IS THE DOMINANT TRANSCRIPTION FACTOR REGULATING THE OA PROGRESSION

The FoxO (Forkhead‐box class O) transcription factor family for mammals mainly includes FoxO1, FoxO3, FoxO4 and FoxO6, which perform distinct and overlapping functions. The domain of its specific DNA binding site comprises three alpha‐helices and two characteristic outer loops,^18^ as the shape of a fork. FoxO is mainly involved in the physiological regulation of biological development and ageing, which is closely related to the lifespan of vertebrates. In addition, FoxO transcription factors are also involved in critical roles such as preventing oxidative stress damage and maintaining homeostasis of the intracellular environment.^19^ Even though FoxO1, FoxO3 and FoxO4 are expressed ubiquitously in multiple organs, the gene regulation of each FoxO conforms to a tissue‐specific pattern, while FoxO6 expression largely correlates to neurological function.^20^, ^21^ FoxO1 is primarily expressed in bone and cartilage and functions as an essential factor in regulating bone tissue development and maintaining chondrocyte homeostasis.^22^ FoxO1 interacts with Runx2^23^, ^24^ and Wnt/β‐catenin cellular pathway in osseous tissue to regulate osteoblast differentiation and further modulates energy metabolism to influence bone development.^22^, ^25^ The longevity of homosapien is associated with the polymorphisms of FoxO3.^26^

FoxOs are the downstream target of phosphoinositide‐3 kinase (PI3K)/Akt signalling, which modulate cell proliferation, growth, apoptosis, and the expression of antioxidant and autophagy proteins.^27^ FoxO controls the cellular homeostasis and the subsistence of stem cells in development to maintain the biological function of the tissue.^27^ FoxOs also regulate the self‐renewal of stem cell populations^28^ and are involved in the regulation of cell differentiation.^29^ Oxidative stress induces the FoxOs expression and transcription. The downstream antioxidant enzymes, including catalase and manganese superoxide dismutase, are activated to prevent impairment of cellular homeostasis.^30^ Besides, autophagy and ubiquitin‐proteasome system are the two primary intracellular clearance mechanisms regulated by the transcriptional function of FoxOs. Recycling and eliminating damaged organelle and dysfunctioned protein are significant for cellular homeostasis.

FoxO‐meditated autophagy has been implicated as the essential regulator for chondrocytes homeostasis preventing the intervertebral disc and articular cartilage from degradation.^19^, ^31^ FoxO1 expression decreases in the cartilage of elderly OA patients, especially in the superficial layer of the cartilage in the weight‐bearing area. Similarly, in the fibrosis area and osteophytes hyperplasia area, FoxO1 presents a significantly lower expression, which reveals the phenomenal correlation between OA progression and FoxO1 expression.^22^ Animal experiments reveal that FoxO1 expression gradually decreased with increased mouse age and the chondrocytes with lower FoxO1 expression aggregated in the cartilage not covered by the meniscus. The accumulation of microdamage induced by biomechanical stress on the cartilage regulates the FoxO1 expression and OA progression.^31^ Apart from cartilage degradation, the meniscus damage accounted for the primary factor for knee OA. FoxO1 and FoxO3 are more abundant than FoxO4 in human menisci, and FoxO1 and FoxO3 show similar expression in the whole meniscus regions. In mature mice, a higher expression of FoxO1 and FoxO3 shows in the meniscus's superficial zone and posterior region. In degenerated meniscus, FoxOs, especially FoxO1 and FoxO3, are depressed, revealing their protective functions during ageing‐induced OA progression.

Ageing is the leading risk factor for meniscus degeneration.^32^ The histological autopsy analysis of human knee joints reveals that meniscus from donors with normal articular cartilage presented minimal reduced FoxO1 or FoxO3 expression.^33^ On the contrary, mice showed ageing‐related meniscus damage and lowered FoxO1 and FoxO3 expression at 12‐month age,^34^ where FoxO1 and FoxO3 expressions decreased in the posterior vascular zone. Similar to the ageing‐induced OA model, reduced FoxO expression correlates to meniscus degradation in DMM and treadmill‐induced OA. Col2a1 and Acan aggregated in the superficial and avascular area of the meniscus, prominently in the inner third.^35^, ^36^ Besides, Col2a1 expression is relatively extensive in fibrochondrocytes of anterior and posterior meniscus horns.^37^ The Acan‐CreERT2 FoxO TKO mice spontaneously developed more severe histopathological changes than the meniscus in control mice by 9 months, and treadmill running‐induced meniscus damage was also more stringent. The AcanCreERT2‐FoxO TKO mice show that FoxO is indispensable for the subsistence of meniscus integrity during adulthood, where cultured human meniscus cells with FoxO1 overexpression reveal similar results. FoxO1 regulates chondrocytes viability and cartilage homeostasis, including autophagy, ECM, transcription factors, antioxidant defence, ECM degradation and inflammation. In the meniscus from arthrotrauma, compromised autophagy and suppression of FoxO reveal the protective mechanism against chondrocyte deterioration.^38^ The critical articular lubricant, PRG4, is also regulated by FoxO1 to prevent damage from mechanical stress.^39^ PRG4 is the secretion from the synovium chondrocytes from the superficial zone of articular cartilage and meniscus. In FoxO KO mice, PRG4 presents the lower expression, causing the cartilage to be more susceptible to mechanical stress damage. The chondrocytes in FoxO‐deficient animal models are characterized with hypertrophic morphology, associated with increased abnormal expression of hypertrophy‐related genes. In this regard, FoxO suppression in the chondrocytes might contribute to cartilage degradation and calcification, cellular autophagy impairment and PRG4 decrease, leading to OA's pathogenesis.^40^

## 4 FoxO1‐MEDITATED CELLULAR AUTOPHAGY MAINTAINS THE HOMEOSTASIS OF CHONDROCYTES

**FIGURE 2:**
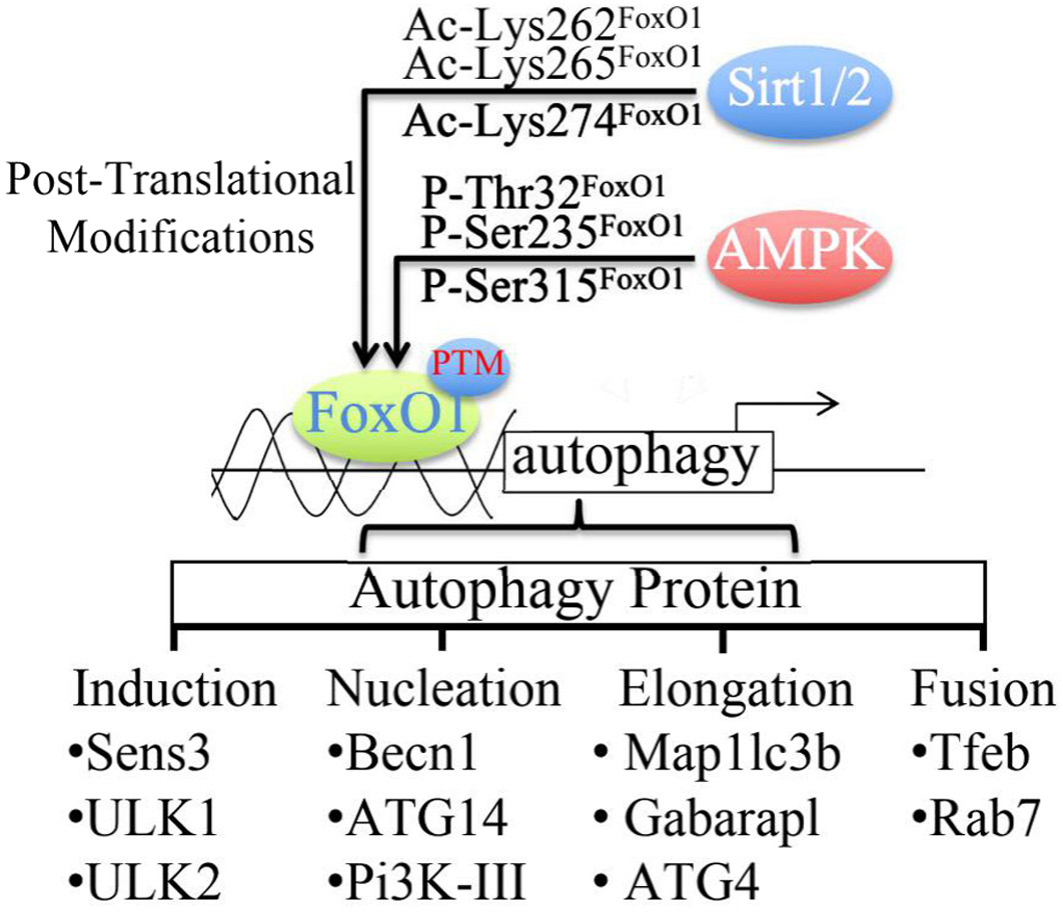
PTM regulates the activation of FoxO1 at specific sites via Sirt1/2 and AMPK, promoting the binding between the FoxO1 and autophagy‐related proteins promoters. FoxO1 is essential in each process of autophagy, including induction, autophagosome nucleation (Nucleation), autophagosome membrane elongation (Elongation) and autophagolysosome fusion (Fusion)

FoxO1 is the dominant downstream target in the PI3K‐Akt kinase pathway and AMPK‐JNK kinase signalling pathway, generally involved in critical physiological functions such as apoptosis, energy metabolism, cell cycle and differentiation. FoxO binds to the gene promoter and up‐regulates the expression of ATGs genes to activate the downstream autophagy initiation.^41^ After the transcription and translation of FoxO from the nucleus into the cytosolic compartment, FoxO undergoes various post‐translational modifications (PTM) such as AMPK phosphorylation, Sirt1/2 acetylation or PRMT6 methylation,^42^, ^43^ which promotes FoxO translocation to the nucleus^41^, ^44^ and activates autophagy initiation. Among all, Sirt1/2‐induced acetylation is indispensable in regulating FoxO1 activity. Sirt1/2 acetylates FoxO1 transcription factor at Lys262, Lys265 and Lys274 in human FoxO1, and AMPK pathway phosphorylates at Thr24, Ser256 and Ser319 in mouse FoxO1, or Thr32, Ser253 and Ser315 in human FoxO1. PTMs can effectively activate multiple phases in the process of autophagy, including phagophore induction(e.g. Ulk1 and Ulk2), nucleation(e.g. Becn1 and Atg14), elongation (e.g. Map1lc3b, Gabarapl and Atg4) and fusion of autophagosomes and lysosomes (e.g. Tfeb, Rab7)^45^, ^46^, ^47^, ^48^ (Figure 2). Additionally, FoxO transcription factors activate mTORC1 by inducing sestrin 3 (Sesn3), the promotor of AMPK and deactivator of mTORC1, eliminating the inhibition of autophagy initiation.^49^, ^50^, ^51^ In general, the mechanism of autophagy regulation is complicated and involved in various signalling pathways. Therefore, the elaboration of the FoxO1 pathway has a fundamental clinical significance for OA pathogenesis.

Studies have revealed multiple cellular pathways regulating the initiation of autophagy and the process of autophagosome formation. ①AMP‐activated protein kinase (AMPK) stimulates the FoxO transcription and induces the ATGs activation to function as the dominant regulator of the autophagy activation pathway. Besides, the ② beclin‐1 complex and ③ serine/threonine‐protein kinase 1 (Unc‐51 like autophagy activating kinase, ULK1) coordinate the activation of autophagy as well. The mTOR signalling pathway is mainly involved in inhibiting autophagy.^52^, ^53^ AMPK alteration is sensitive to various cell oxidative stress injuries. The activation of kinase in AMPK promotes the downstream target phosphorylation, including ULK1 and mTORC1, acting as the initiator of autophagy.^54^ The ULK1 complex, which consists of ULK1, ATG13, ATG101 and FIP200, positively regulates autophagy resulting in the initiation of autophagosome formation. AMPK‐induced phosphorylation of ULK1 itself promotes the early‐stage autophagosome formation.^55^ The autophagosome initiation elevates the aggregation of other ATGs proteins to accelerate the elongation and maturation of the autophagosome. ATG7 is the dominant protein regulating the autophagosome initiation, which functions as the ubiquitin‐like modifier‐activating enzyme. ATG5 is ubiquitylated by ATG7 and combined with ATG12 to form a functional complex.^56^ This protein complex acts as an E3‑ubiquitin‐like ligase, promoting the lipidation of the cytoplasmic form of microtubule‐associated proteins 1A/1B light chain 3A (LC3), known as LC3‑I, and converting it to the membrane‐bound phosphatidylethanolamine‐conjugated format, known as LC3‑II.^57^ The structural alteration of LC3‑I to LC3‑II is the fundamental of autophagosome maturation. The conversion of LC3‑I to LC3‑II is an essential process in the maturation of autophagosomes, which facilitates the amalgamation of lysosomes for substance degradation and recycling.^58^ In the autophagy formation process, the beclin‐1 complex is regulated by phosphatidylinositol 3‐kinase (PI3K) and binds to the inner membrane structure in the cell (endoplasmic reticulum, mitochondria or nuclear membrane), promoting the ULK1 complex to forms the outer membrane structure of the autophagosomes. The colonization of ATG14L facilitates the transport and maturation of autophagosomes. On the contrary, ULK1 can also negatively regulate AMPK and deactivate autophagy. The mTOR can phosphorylate ATG13 to inhibit the outer membrane formation for the ULK1 complex.^11^

The impacts of FoxO1 phosphorylation on autophagy are significantly different in various pathways and cells. Phosphorylation induced by AKT,^59^ ERK,^60^ MEK^61^ and AMPK is the essential PTMs of Foxo1 that trigger the alteration of subcellular localization.^62^ Phosphorylated‐Foxo1(p‐FoxO1) is restricted in the cytoplasm and interacts with ubiquitin E3 ligases, evoking the degeneration of Foxo1. Furthermore, p‐FoxO1 binding to 14‐3‐3 protein extends the physical distance of FoxO1 and downstream target, preventing the binding and activation of targeted DNA from transcription.^63^, ^64^ That ERK cellular pathway is one of the critical signals of FoxO1 phosphorylation. The inhibition of ERK and MEK attenuate phosphorylation resulting in the activation of Foxo1 in rat hepatocytes.^61^ Phosphorylation of FoxO1 is invalid in binding to ATG7 for autophagy initiation in mouse ovarian granulosa cells. The FSH or melatonin induces both Akt‐mediated phosphorylation and Sirt1‐mediated deacetylation of FoxO1. However, neither FoxO1‐ATG7 interaction nor autophagy upregulation was verified.^42^, ^45^ Interestingly, a recent study revealed a specific result that phosphorylated cytosolic FoxO1 associates with Atg7 to induce autophagy in iNKs, which is essential for NK cell development. Modified FoxO1 promotes the interaction with ATG7 and stabilizes FoxO1 in iNKs cytoplasm, leading to the autophagy initiation.^43^ A comparable study has suggested that Akt‐induced FoxO1 phosphorylation excludes FoxO1 from the nucleus to cytoplasm, resulting in the enhancement of FoxO1–Atg7 interaction and promoting autophagy activation in the cytoplasm.^43^, ^65^

The AMPK‐FoxO–autophagy pathway contributes to cell viability with the essential adaptation mechanism, maintaining cellular homeostasis from environmental stresses, primarily protecting chondrocytes from exercise‐induced hypoxia and microdamage.^66^ AMPK induces FoxO3 phosphorylation at Ser413 or Ser588 and stabilizes the activation in nutrient deficient or hypoxia situations. The nucleus accumulation of p‐FoxO3 significantly increases autophagy gene transcription and promotes autophagy via transcriptional or epigenetic mechanisms.^41^, ^44^, ^66^, ^67^ Likewise, FoxO3 is phosphorylated by c‐Jun N‐terminal kinase (JNK) at Ser294, inducing nuclear translocation under oxidative stress. JNK‐FoxO3‐autophagy pathway plays a critical role in bone remodelling. By elevating the mitochondrial metabolism, the JNK‐FoxO3‐autophagy pathway promotes the differentiation of mesenchymal stem cells into osteoblasts, which is vital for bone homeostasis.^68^ FoxO1 is proved to be indirectly phosphorylated by JNK at Ser246 through cyclin‐dependent protein kinase (CDK) in neurons^69^ and activates AMPK via SESN to promote autophagy activation.^50^, ^51^

**FIGURE 3:**
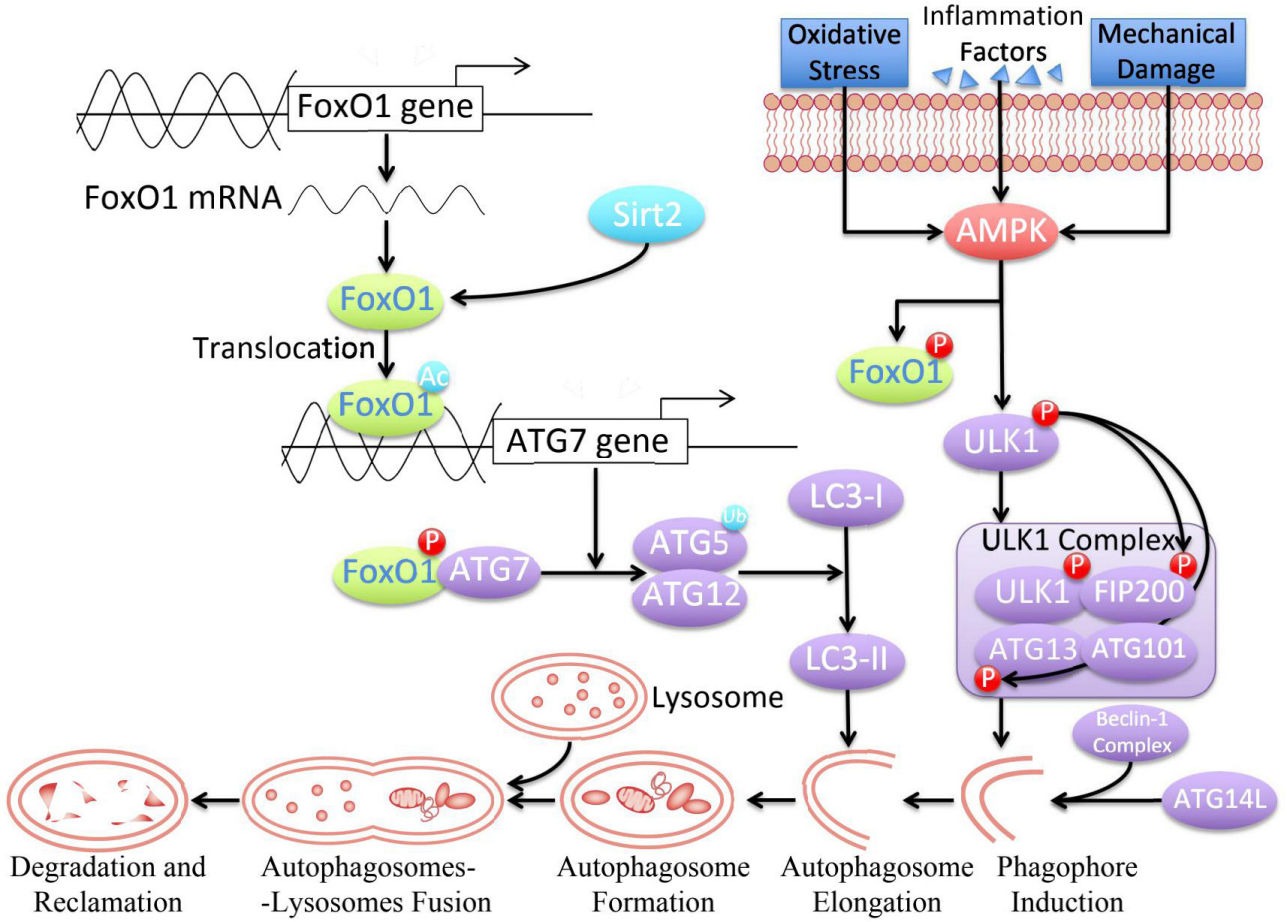
Activation process of autophagy regulated by FoxO1. In response to the stimulation of various harmful factors, AMPK activates the FoxO1 phosphorylation and formation of the ULK1 complex, which initiates autophagy with Beclin‐1 complex, ATG14L and other molecules. In the elongation phase of the autophagosome formation, the transformation of LC3‐I to LC3‐II is essential for continuing the autophagy process. Acetylated FoxO1 can regulate the expression of ATG7. Meanwhile, phosphorylated FoxO1 form a FoxO1‐ATG7 complex, activate the ubiquitinase activity of ATG7 and perform ubiquitination on the ATG5‐ATG12 protein complex

Apart from binding to the promotors to upregulate the expression of autophagy‐related genes, FoxOs independently activate autophagy without transactivation functions.^45^ FoxOs translocate from the nucleus to the cytosolic compartment and combine directly to the E1‐like enzyme, ATG7, to elevate autophagy activity. The acetylation (e.g. Lys262, Lys265 and Lys274 in Homo sapiens FoxO1)^45^, ^70^ and Akt‐induced phosphorylation (e.g. Thr24, Ser256 and Ser319 for Mus musculus Foxo1, and Thr32, Ser253 and Ser315 for H. sapiens FoxO1)^43^ are critical for FoxO1 translocation into the cytoplasm and the Atg7 interaction for autophagy induction. Stress conditions promoted the dissociation of deacetylases Sirt1 or Sirt2 from their substrate FoxO1, which significantly upregulated the acetylation of FoxO1.^71^ SIRT1 functions as the autophagy initiator primarily in the nucleus,^72^ while SIRT2 acts as the dominant deacetylase of FoxO1 primarily in the cytosol.^73^, ^74^ Three acetylation sites of FoxO1 (Lys 262, Lys 265 and Lys 274) have been identified as the functional target of SIRT2. Deacetylated FoxO1 can promote the activation of autophagy. In 3T3‐L1 preadipocytes, a recent study has proved the indirect interaction of SIRT2 and FoxO1. In response to stimuli, the release of SIRT2 from FoxO1 induces acetylation and promotes its transactivation,^75^, ^76^ and cytosolic FoxO1 is primarily accountable for the initiation of autophagy. In human colon tumours, the acetylated FoxO1 and ATG7 complex functions as the dominant initiator for autophagosome formation (Figure 3). ATG7 functions as the E1‐like protein to activate ATG12 by forming a thioester bond between Cys 507 of Atg7 and Gly 186 of Atg12. The activation of ATG12 by ATG7 is indispensable for inducing ATG12‐ATG5 conjugates and membrane‐bound LC3‐II in mammalian cells.^77^ The acetylation of FoxO1 is essential for autophagy initiation. However, enhancing ATG7 activity after the interaction with acetylated FoxO1 still needs detailed elaboration. The physiological mechanism of the FoxO1 signalling pathway in chondrocytes requires specific exposition. This review discussed, at least in part, how the FoxO1 correlated with autophagy initiation and the protective ability in cartilage and bone homeostasis.

## CONFLICT OF INTERESTS

All authors declare that they have no conflict of interest.

## AUTHOR CONTRIBUTION

**Jiaji Yue:**Funding acquisition (lead); Writing – original draft (lead). **Weichao Sun:** Visualization (equal). **Aikebaier Aobulikasimu:** Writing – original draft (supporting). **Shuyu Liu:** Visualization (equal). **Wei Xie:** Writing – review & editing (supporting). **Wei Sun:** Writing – review & editing (equal).

## ETHICAL APPROVAL

The ethical approved was exempted by the institutional review board of Shenzhen Second People's Hospital.
